# PytuTester: RaspberryPi open-source ventilator tester

**DOI:** 10.1016/j.ohx.2022.e00334

**Published:** 2022-06-30

**Authors:** Félix Morales, Luis Bernal, Gustavo Pereira, Sandra Pérez-Buitrago, Michael Kammer, D.H. Stalder

**Affiliations:** a Facultad de Ingeniería, Universidad Nacional de Asunción, Paraguay; b Departamento de Ingeniería, Pontificia Universidad Católica del Perú, Peru; c Division of Allergy, Pulmonary, and Critical Care Medicine, Vanderbilt University Medical Center, Nashville, TN 37235, United States

**Keywords:** Ventilator tester, Low-cost, Open source, Medical hardware, Pandemic COVID-19

## Abstract

PytuTester is an open-source ventilator tester developed to help bio-engineers in the design and verification of new ventilator prototypes. A ventilator tester allows measuring the flow, pressure, volume, and oxygen concentration provided to the patient. During the global pandemic COVID-19, several open-source ventilators prototypes were developed; however, due to high cost and demand testers, they were not available. In this context, a low-cost tester was developed using a Raspberry Pi and medical-grade sensors for the test ventilators prototypes. This paper presents the design files, software interface, and validations tests. Our results indicate that the tester has good accuracy to evaluate the efficacy and performance of new prototypes. When tested on two ventilator designs developed in Paraguay, PytuTester reported flow profiles that were concordant with the industry-standard VT650 Gas Flow Analyzer. PytuTester was then field deployed to test several DIY ventilator designs in low-resource areas.

## Graphical abstract

**Figure:**
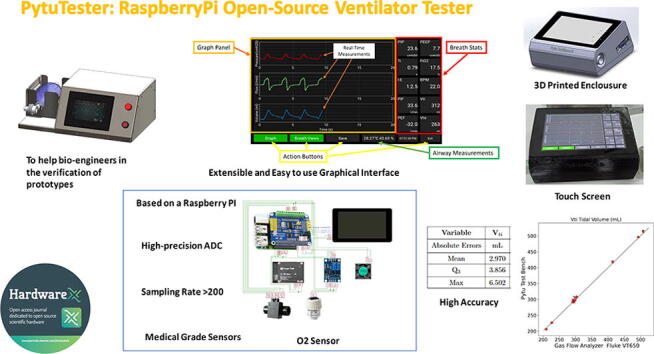

**Specifications table**

**Table:** 

**Hardware name**	*PytuTester*
**Subject area**	*Medical*
**Hardware type**	*Medical hardware*
**Open-source license**	*CERN-OHL*
**Cost of hardware**	*USD 500*
**Source file repository**	Mendeley Data: *https://doi.org/10.17632/w6v48k72z8.1.*

## Hardware in context

The coronavirus disease (COVID-19) pandemic was declared on March 11, 2020. By August, 2021, COVID 19 had caused more than 4 million deaths worldwide and more than 200 million infections [1]. The pandemic has generated a crisis in health systems due to the lack of human resources, medical equipment, medical devices, and sanitary protection. Since COVID-19 affects the respiratory system, ventilators and other devices intended for respiratory support were highly demanded. It was extremely difficult to obtain medical equipment and supplies because most countries prohibited the medical industry from selling outside of their own countries [2]. As a consequence, bio-engineers and medical experts in mechanical ventilation worldwide volunteered to develop open-source ventilator designs for emergency use [3]. However, any ventilator must pass several verifications and validation steps according to applicable standards; such as, Specification for Rapidly Manufactured Ventilator System (RMVS) or ISO 80601-2-12 [4], [5]. This process requires a ventilator tester to evaluate its performance and efficacy. Commercially available ventilator testers, including the Fluke VT650/900, TSI 4070/4080 Certifier, Rigel Ventest, and CITREX H3/4/5, are costly (USD 5,000 – USD 12,000), and difficult to obtain in low-resource settings [6], [7], [8]. Additionally, several gas flow sensors do not provide adequate performance: Fluke gas flow analyzers only allow saving data with a maximum of 1 Hz sampling rate, which is insufficient to perform detailed comparison and post-processing analysis of each test [6].

Several open-source testers have been developed during the pandemic, eg. VISP (Ventilator Inline Sensor Package), VentMon, and COVENT-Tester, [9], [10], [11] (see Table 1). VISP employs three general use pressure sensors, but the project awaits validation. VentMon introduces the use of a low-cost medical-grade digital flow sensor. COVENT-Tester introduces analog medical-grade flow and oxygen sensors. However, according to standards RMVS and ISO 80601-2-12, at least 200 samples per second are required.

**Table 1:** Open-source Testers.

**Features**	**VISP**	**VenMon**	**COVENT-Tester**	**PytuTester**
Medical Sensors	No	No	Yes	Yes
Sampling Rate	<100 sps	<100 sps	100 sps	>200 sps
GUI	OLED	No	OLED $0.96^{\prime \prime }$	LED $7^{\prime \prime }$
Battery	No	No	No	5000 mAh
Cost(USD)	20	280	190	530

This paper, therefore, presents a new open-source ventilator tester, “PytuTester”. This design can measure parameters such as flow, airway pressure, and oxygen concentration with a high sampling rate. PytuTester was designed based on maximizing affordability, portability, and accuracy of measurements First, we present the hardware requirements and description; all schematics, design files, construction material, and software are provided. Then, we present the validation results where the device was tested utilizing a precision test lung and a calibrated gas flow analyzer.

## Hardware description

**Fig. 1:**
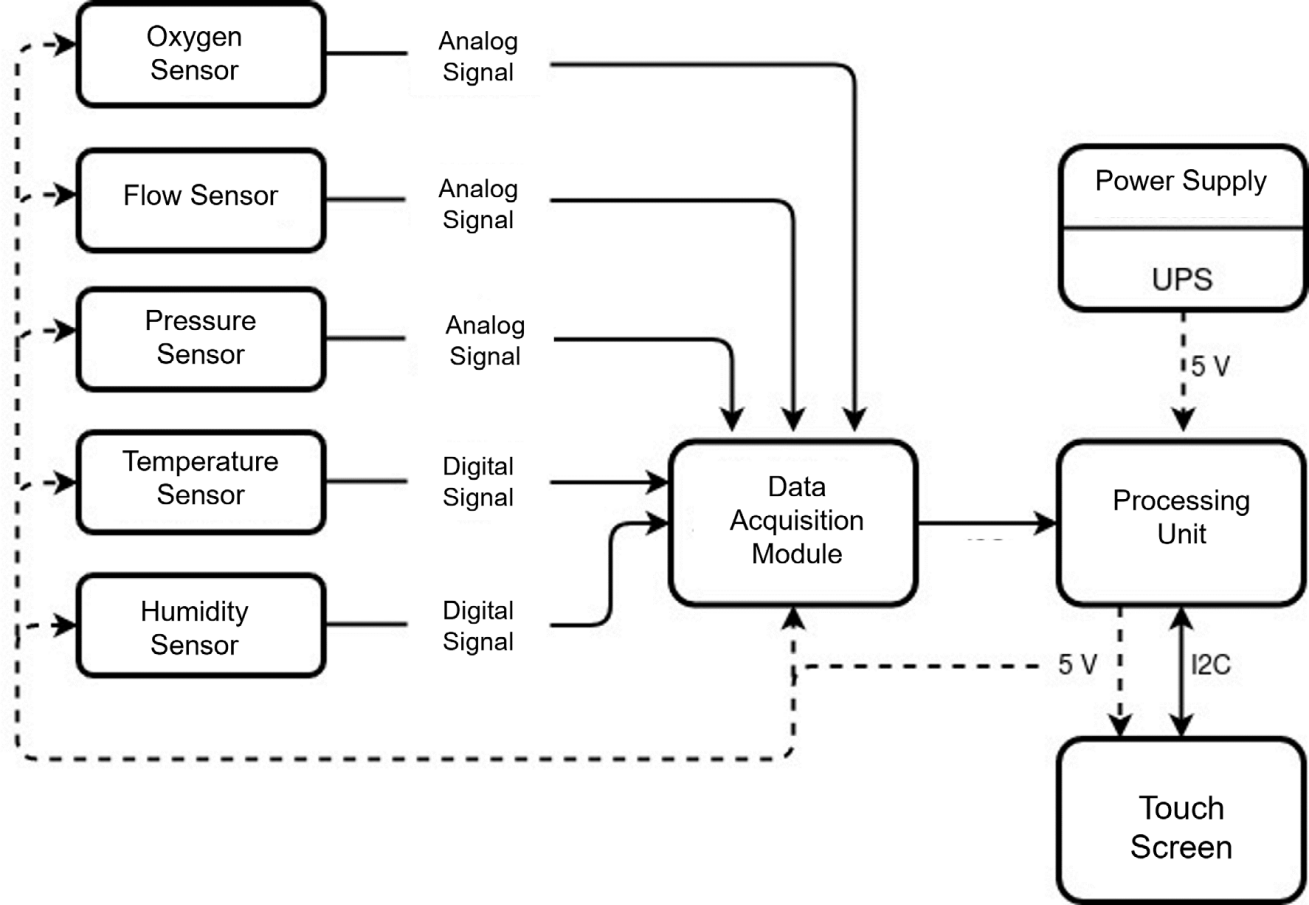
PytuTester block diagram.

PytuTester hardware is composed of medical-grade sensors, a data acquisition module (DAQ), a processing unit (a Raspberry Pi 4), a battery power supply, and a touch screen (see Fig. 1). The processing unit directly reads the sensors with the DAQ. The software sets up the operation mode according to user input through its user interface. There are two operation modes: real-time view to inspect waveforms and data logging mode to perform a postprocessing error analysis.

PytuTester is a low-cost open-source tester dedicated to performing verification tests on medical ventilators prototypes. This device allows real-time visualization and monitoring of the most relevant values to measure ventilator performance, as well as record all relevant data and perform post hoc exhaustive analysis. The primary flow sensor in PyTu is a bi-directional medical respiratory sensor capable of measuring the gas flow rate, gauge pressure, temperature, and humidity of the respiratory circuit. It also incorporates an oxygen concentration sensor, an analog-to-digital converter module, a Raspberry Pi 4, a 7-inch touch screen, a power supply module, and a 3D printed case.

### Sensors

Continuous airflow measurements during pre-clinical tests provide important information for the assessment of prototypes. Our sensing systems comprise two sensors: a respiratory multi-sensor (the Siargo FS6122 Series) and an oxygen sensor (Sensoronics SS-08). The FS6122 sensor has two analog outputs (pressure and flow rate) and a digital interface via Inter-Integrated Circuit (I^2^C) communication. The flow and pressure can be measured from −250 to 250 SLPM (with an accuracy of ± (2.5 + 0.5FS)%) and from −5 to 100 cmH_2_O (with an overall accuracy corresponding to 1.0 %FS), respectively. The analog outputs have a range from 0.5 V to 4.5 V. The temperature and relative humidity are read via I^2^C.

The electro-galvanic oxygen sensor measures the oxygen concentration in a range of 0 to 100% O_2_. The output is an analog voltage between 8.0 mV and 15 mV for a 20.9% O_2_ of oxygen concentration, with a response time of 7 s according to ISO/DIS 7767. However, due to the small output range produced by this sensor, it is necessary to include an instrumentation amplifier to obtain a signal in the data acquisition input range. The module AD620 provides a platform for amplification of small signals from analog sensors with a gain between 1.5× and 1000×.

### Processing unit

The processing unit must allow control over the sampling process and the user interface. For this reason, a Raspberry PI 4 was selected to meet these requirements at a reasonable cost. Its relevant processing power is allied to the simplicity of implementation with the following technical specifications: Broadcom BCM2837, a 64-bit Quad-Core 1.5 GHz CPU, 4 GB RAM, Ethernet 10/100 Wireless 2.4 GHz 802.11n, 5 V with 3.8 W to 4.0 W, microSD, GPIO, I^2^C, SPI, HDMI, Audio-video (3.5 mm), 4 x USB 2.0.

### Data acquisition

The ADCs (Analog to Digital Converters) allow digital computers to interact with analog sensors signals, thus performing their sampling and quantization. An important consideration is the maximum resolution and theoretical error of an ADC: by adding up values of parameters such as offset error, gain error, noise, and non-linearity integral error, we will have a total value of maximum theoretical error that directly affects the precision and accuracy of the readings, and this error should also be considered when choosing the most appropriate AD converter. Considering these limitations, a Wave Share high-precision analog-to-digital converter module was selected to read the sensor’s analog signals. This expansion module facilitates addition of the high-precision AD/DA functions to the Raspberry Pi. The Wave Share ADC integrates the ADS1256 converter, which enables 8 channels with a resolution of up to 24 bits and a sampling rate ranging from 2.5 to 30000 samples per second, in an input range from 0 to 5 V.

### Screen and power supply

PytuTester has a touchscreen display that shows multiple measurements at once and quickly controls several options modes described in the next subsection. It can display real-time measurements with either color graphs or numerical data. The $7^{\prime \prime }$ touchscreen gives the ability to create an all-in-one embedded GUI with 800 × 480-pixel resolution. This display is compatible with a Raspberry Pi via an adapter board that handles power and signal conversion. Only two connections to the Pi are required, the power from the GPIO port and a ribbon cable that connects to the DSI port present on all Raspberry Pi Model B boards. It has outer dimensions: 192.96 × 110.76 mm, a visible area: 154.08 × 85.92 mm. It consumes only 200 mA at 5 V with maximum brightness.

### Power supply

The power supply provides a voltage of 5 V and up to 3.0 A to the system when connected to the power outlet. However, since the equipment needs to be used in situations where power outages do not interfere with the procedures and also make portable use possible, an uninterruptible power supply (UPS) module was incorporated. The PiSugar 2 Pro module has been selected to provide the device with the necessary backup power. This portable power platform is specially designed for Raspberry Pi with a 5000 mAh battery which can last for 8–10 h. It integrates UPS, RTC, I2C battery management, and custom buttons.

### Firmware

The main firmware is written in the python 3 environment using an open-source multi-platform GUI development library called Kivy.^1^ This python application uses another library called PiPyADC^2^ for interfacing Texas Instruments SPI bus-based A/D converters with the Raspberry Pi. This module manages reading the various sensors and renders it in a graphical display appropriate for bioengineers.

**Fig. 2:**
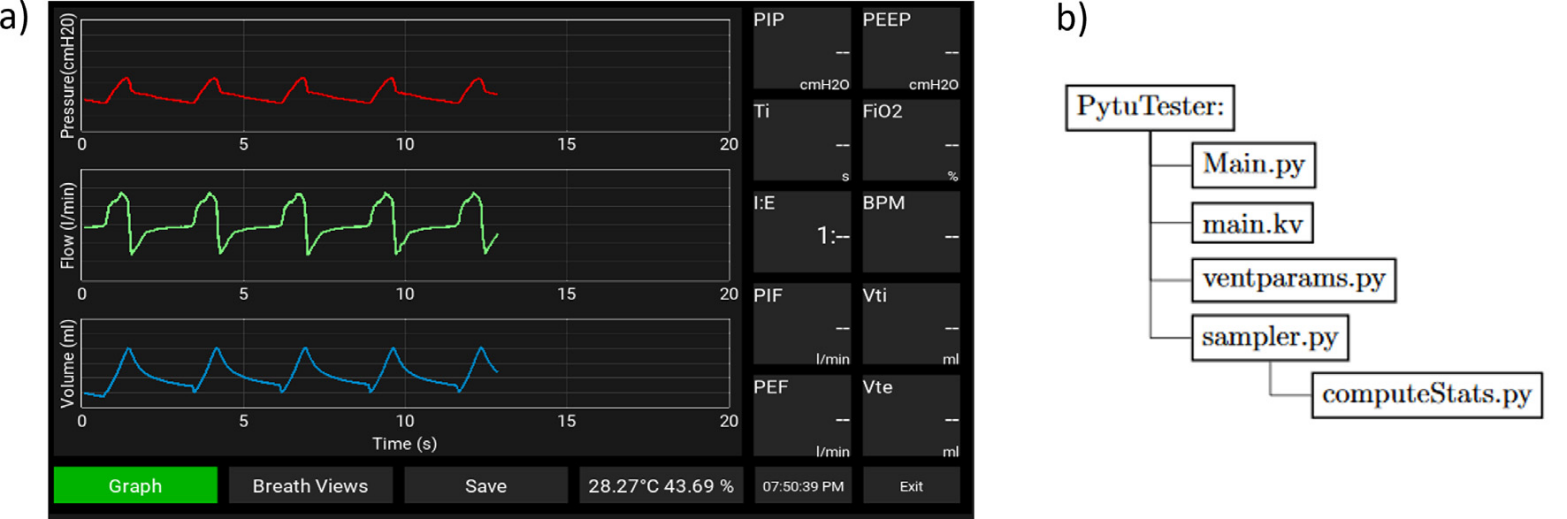
(a) Graphical User Interface, (b) Software Organization.

This PytuTester has two modes of operation: (A) A real-time mode to show the pressure, flow, and volume graphs and (B) Data logging mode to save raw pressure and flow values with a high sampling rate. Mode (A) also have another option to compute (for each respiratory cycle) breathing stats such as the tidal volume ($V_{\mathrm{ti}}$), the respiratory frequency (BPM), the inspiratory time ($T_{i}$) and the expiratory time ($T_{e}$), inspiration to expiration ratio (IE), the peak inspiratory pressure (PIP), the positive end-expiratory pressure (PEEP), and oxygen concentration FiO_2_. Mode (B) the processing unit is responsible for storing the sensor’s raw data in the SD card with a high sampling rate (>200) so that another script can compute breath stats during post-processing of the data. Fig. 2 shows the Graphical User Interface (GUI) developed to control the operation modes with the touch screen.

**Fig. 3:**
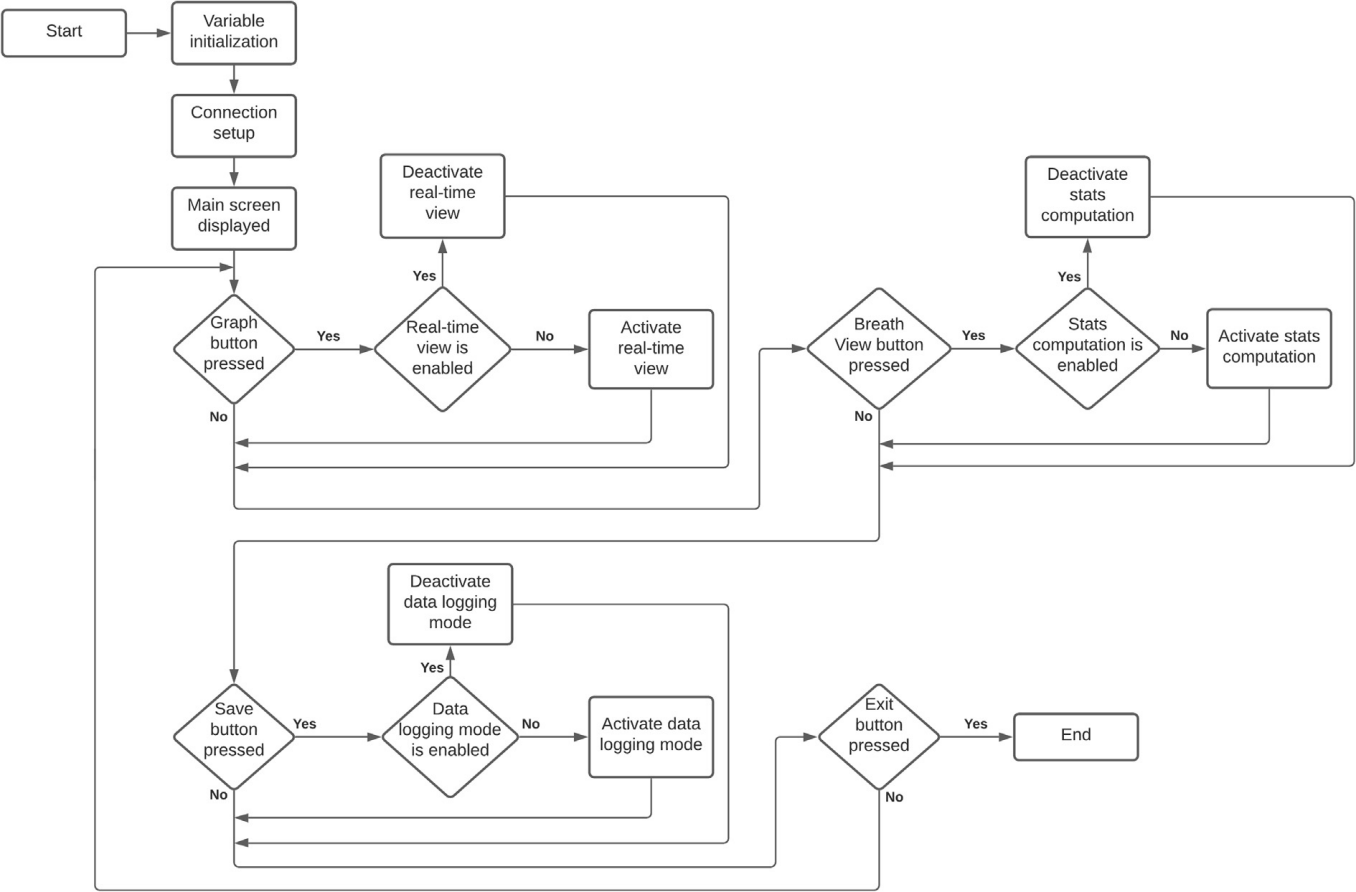
PytuTester main algorithm.

The flowchart that describes the main algorithm implemented is presented in Fig. 3. Once the unit is started, the variables used within the program are initialized. Afterward, the analog-to-digital converter module is configured by adjusting the sampling rate and resolution required by the application. Also, the digital communication addresses for temperature and humidity reading are configured. From there, the main menu appears on the screen, showing the layout of the graphs, the breathing stats, and the selection buttons. The temperature and humidity of the airway are displayed at the bottom, which has an update time of 10 s. The time is also visible in this menu. The refresh rate of this window is set to 25 frames per second.

Enabling the visualization and statistics to be displayed depends essentially on the selection of the operator through the options reflected in the buttons. Therefore, the flow of the process is determined by the selection of the modes belonging to the real-time view, the computation of breathing stats, and data logging.

### Device usefulness potential

This new tester can be useful to build capacity in biomedical engineering, particularly in low-resource settings where commercial ventilator testers are in low supply. Therefore, the benefits and advantages are outlined as:

• The hardware and software are completely open-source.
• The battery and its 3d structure guaranteed its portability.
• The software implements an extensible graphical interface.
• It has medical-grade sensors.
• The DAQ has a configurable sampling rate (up to 30 ksps).
• The configurable operation modes facilitate multiple analyses.

## *Design files*

We provide all design files for the 3D printed parts, the circuit schematics, and software with their corresponding link to online repositories.

## Design files summary

See Table 2.

**Table 2:** Design files list. All files are available in the online repository: https://doi.org/10.17632/w6v48k72z8.1.

**Design filename**	**File type**	**Open-source license**
3D Printed Parts	STL	CERN-OHL

Electronic Schematic	SCH	CERN-OHL

Software	Python 3	CERN-OHL

## Bill of materials summary

See Table 3.

**Table 3:** Bill of materials.

**Designator**	**Component**	**Number**	**Cost per unit (USD)**	**Total cost (USD)**	**Source of materials**	**Material type**
1	Raspberry Pi 4: Model B 4 GB RAM	1	55.00	55.00	Digikey	Semiconductor/ Other
2	7 inch Touch Screen	1	74.89	74.89	Amazon	Semiconductor/ Other
3	Multi-parameter sensor	1	170.00	170.00	Digikey	Semiconductor/ MEMS
4	Oxygen Sensor	1	64.00	64.00	Sensoronics	Semiconductor/ Other
5	ADC/DAC Board	1	33.60	33.60	Amazon	Semiconductor/ Other
6	Portable power platform	1	49.99	49.99	Amazon	Semiconductor/ Other
7	Instrumentation Amplifier Module	1	6.99	6.99	Amazon	Semiconductor/ Other
8	Sink Single Cooling Fan and RAM Heatsink	1	9.99	9.99	Amazon	Semiconductor/ Other
9	Raspberry Pi 4 Power Supply	1	9.99	9.99	Amazon	Semiconductor/ Other
10	micro SD 32 GB	1	7.99	7.99	Amazon	Semiconductor/ Other
11	M2.5 Male Female Hex Brass Spacer Standoff Screw Nut Assortment	1	12.99	12.99	Amazon	Metal
12	Oxygen Sensor T	1	20.88	20.88	Vitatech	Plastic
13	Oxygen Tubing	1	2.99	2.99	Lancetahg	Semiconductor/ Other
14	3D Printer Filament PLA 1.75 mm, total used 302.66 grams	1	7.78	7.78	Aliexpress	Plastic

## Build instructions

PytuTester was designed to be compact, portable, and easy to use for the user, the construction process was divided into three stages: the 3D printed parts, sensors and elements assembly, and the software installation.

### 3D printed parts

**Fig. 4:**
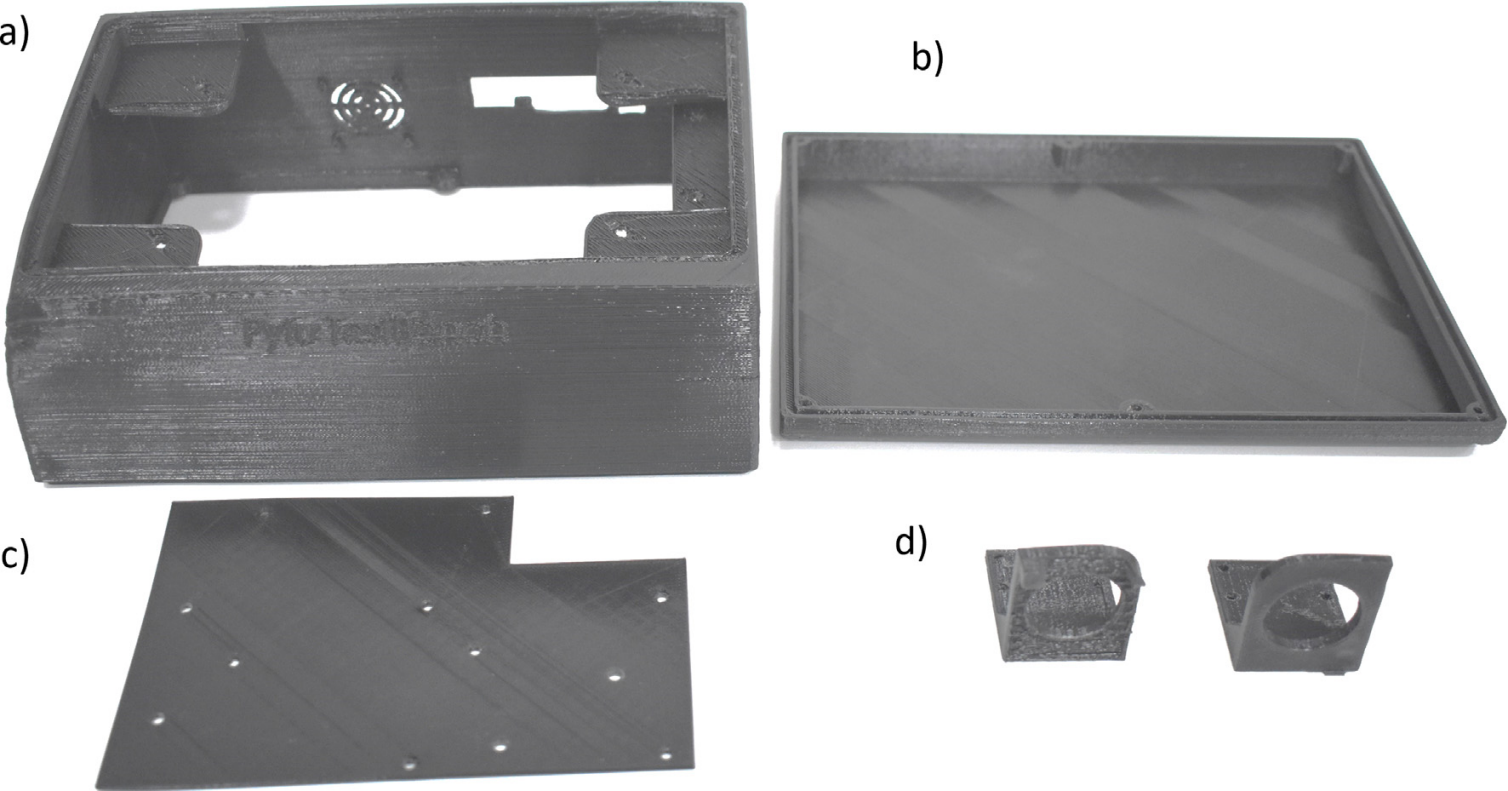
3d Printed Parts. (a) Enclosure top, (b) Enclosure base, (c) PCB separator, (d) Sensors Support.
• is the base of the equipment, it also has two folding supports to improve its ergonomic features. The infill setting of 2% helps to reduce the printing time (Fig. 4. (b))
• PCB Separator: designed to integrate the electronics modules, separate them from the touch screen. The infill setting used was 30% (Fig. 4. (c))
• Multi-sensor holder: custom-designed to fix the FS6122 multisensor concentrically with the outlet hole. The infill setting used was 30% (Fig. 4. (d))
• Oxygen sensor holder: custom-designed to fix the oxygen sensor concentrically to the inlet hole. The infill setting used was 30% (Fig. 4. (d))

All the parts that were designed and printed were made with a 3D printer Prusa, with the layer thickness of 0.2 mm. The material used was PLA with a filament diameter of 1.75 mm, the parts are described below:

• Enclosure top: compact design, in which it was considered that the front face has enough space to incorporate a seven-inch touch screen with an inclination of 10°. It also has a place for the small DC 5 V fan, an inlet hole that was designed to fit the medical T-tube, an outlet hole that was designed to fit the flow sensor, and finally the holes for the connection of peripherals. The infill setting of 2% helps to reduce the printing time (Fig. 4. (a))

### Elements and sensors assembly

The steps needed to assembly all the parts are described below:

1. Place M3 insert nuts on the enclosure top: I1-I6 (Fig. 5. (a)).

**Fig. 5:**
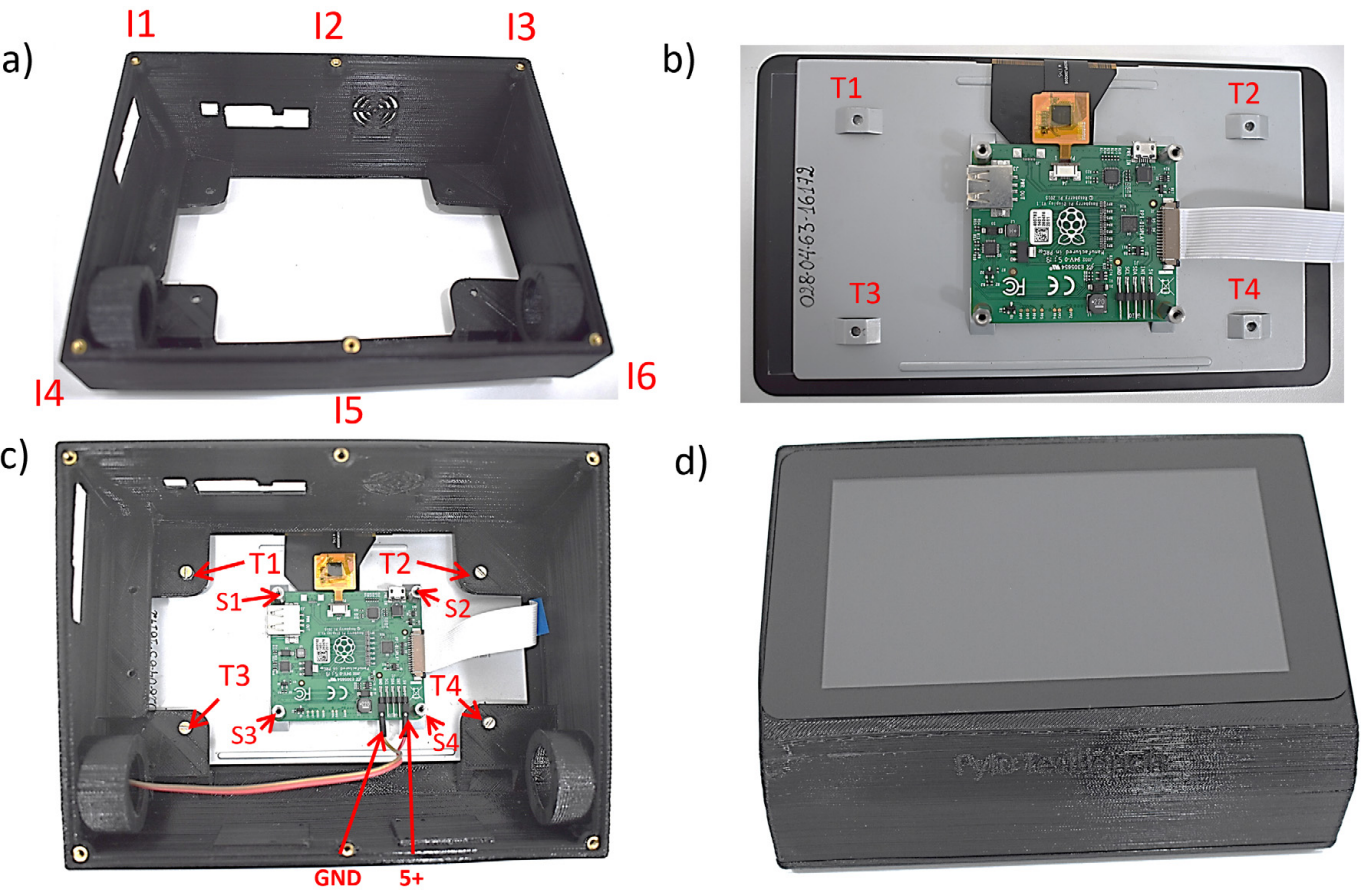
Assembly Part 1: (a) Enclosure top, (b) Display, (c) Assembly, (d) Frontal View.
2. Place the $7^{\prime \prime }$ touch screen in the upper.
3. Tighten with M3 × 6 mm screws: T1-T4 (Fig. 5. (c)).
4. Place male to female hexagonal spacer M2.5 × 6 mm on the display board: S1-S4 (Fig. 5. (c)).
5. Connect the display 5 V power and the ground (Fig. 5. (c)).
6. Place the PCB Separator over the display module, fix with 4 screws M2.5 × 6 mm (T5-T6) and 2 screws M3 × 6 mm (T7-T10). Then place 3 hexagonal spacers M2.5 × 10 + 6 mm: S5-S7. (Fig. 6. (a)).

**Fig. 6:**
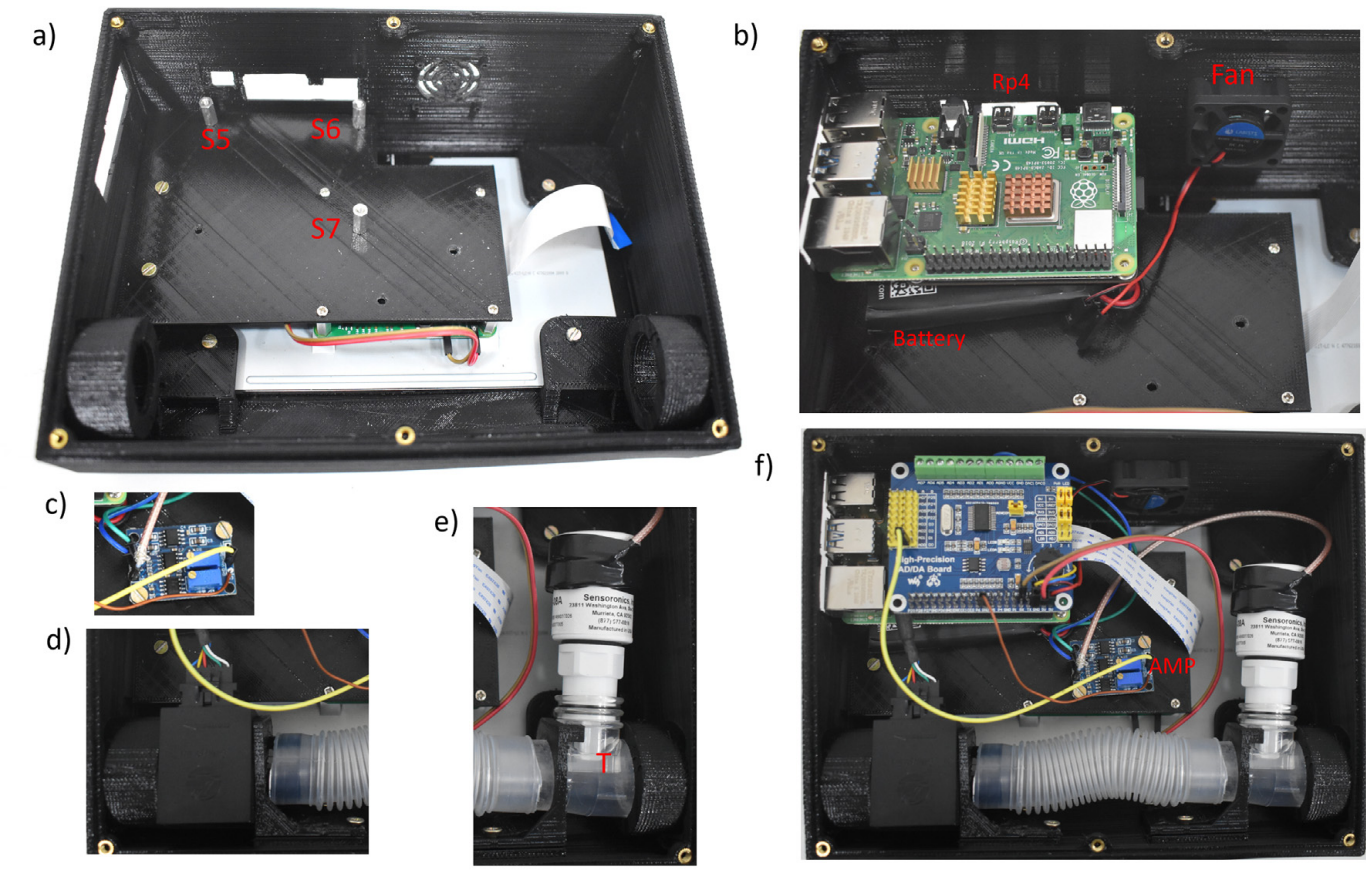
Assembly Part 2: (a) PCB Separator assembly, (b) Raspberry Pi mount, (c) Instrumentation Amplifier, (d) Multisensor, (e) Oxygen Sensor, (f) Final view.
7. Place the oxygen and flow sensors with their corresponding fixing brackets. Use a medical T-tube to connect the oxygen sensor (Fig. 6. (d-e)).
8. Place the PiSugar 2 Pro battery and over it the Raspberry Pi 4. Fix them with 3 M2.5 × 6 mm screws, according to the schematic described in Fig. 7.

**Fig. 7:**
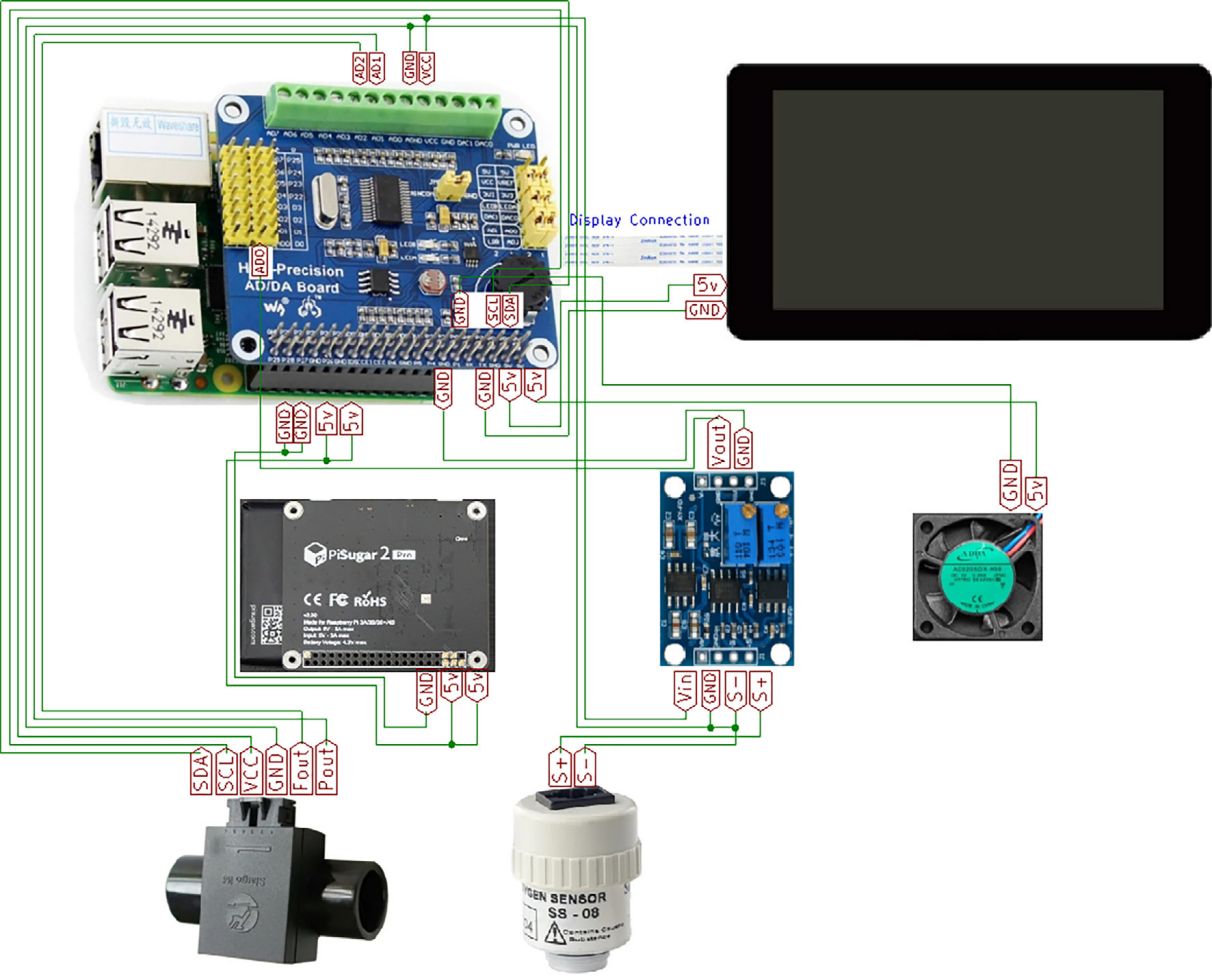
Hardware schematic.
9. Place the fan and its power (Fig. 6. (b)).
10. Place the high-precision ADC module over the Raspberry Pi 4 (Fig. 6. (f)).
11. Place the instrumentation amplifier AD620 using 2 M2.5 × 6 mm screws and connect the oxygen sensor to the input (Fig. 6. (c)).
12. Connect the sensors, the amplifier to the ADC modules, according to the circuit schematic Fig. 7 and Table4.

**Table 4:** Connections.

**UPS**	**Pi 4**	**AD/DA**	**Multisensor**	**Amplifier**	**Oxygen**	**Screen**	**Fan**
5v	Pin 2 (5v)	5v	-	-	-	-	5v
5v	Pin 4 (5v)	5v	-	-	-	5v	-
-	-	VCC	Pin 4 (VCC)	Vin	-	-	-
GND	Pin 6 (GND)	GND	-	GND	-	-	GND
GND	Pin 9 (GND)	GND	-	-	-	GND	-
-	-	AGND	Pin 3 (GND)	GND	-	-	-
-	-	ADO	-	Vout	-	-	-
-	-	AD1	Pin 1 (Pout)	-	-	-	-
-	-	AD2	Pin 2 (Fout)	-	-	-	-
-	-	SCL	Pin 5 (SCL)	-	-	-	-
-	-	SDA	Pin 6 (SDA)	-	-	-	-
-	-	-	-	S+	S+	-	-
-	-	-	-	S-	S-	-	-
-	DSi	-	-	-	-	DSi	-
13. Using an oxygen blender adjust the amplifier gain considering at least two cases, by setting the FiO_2_ to 21% and 100%.
14. Finally, the prototype is closed with the enclosure base and fixed using 6 M3 × 6 mm screws. Fig. 8 shows the final assembly.

**Fig. 8:**
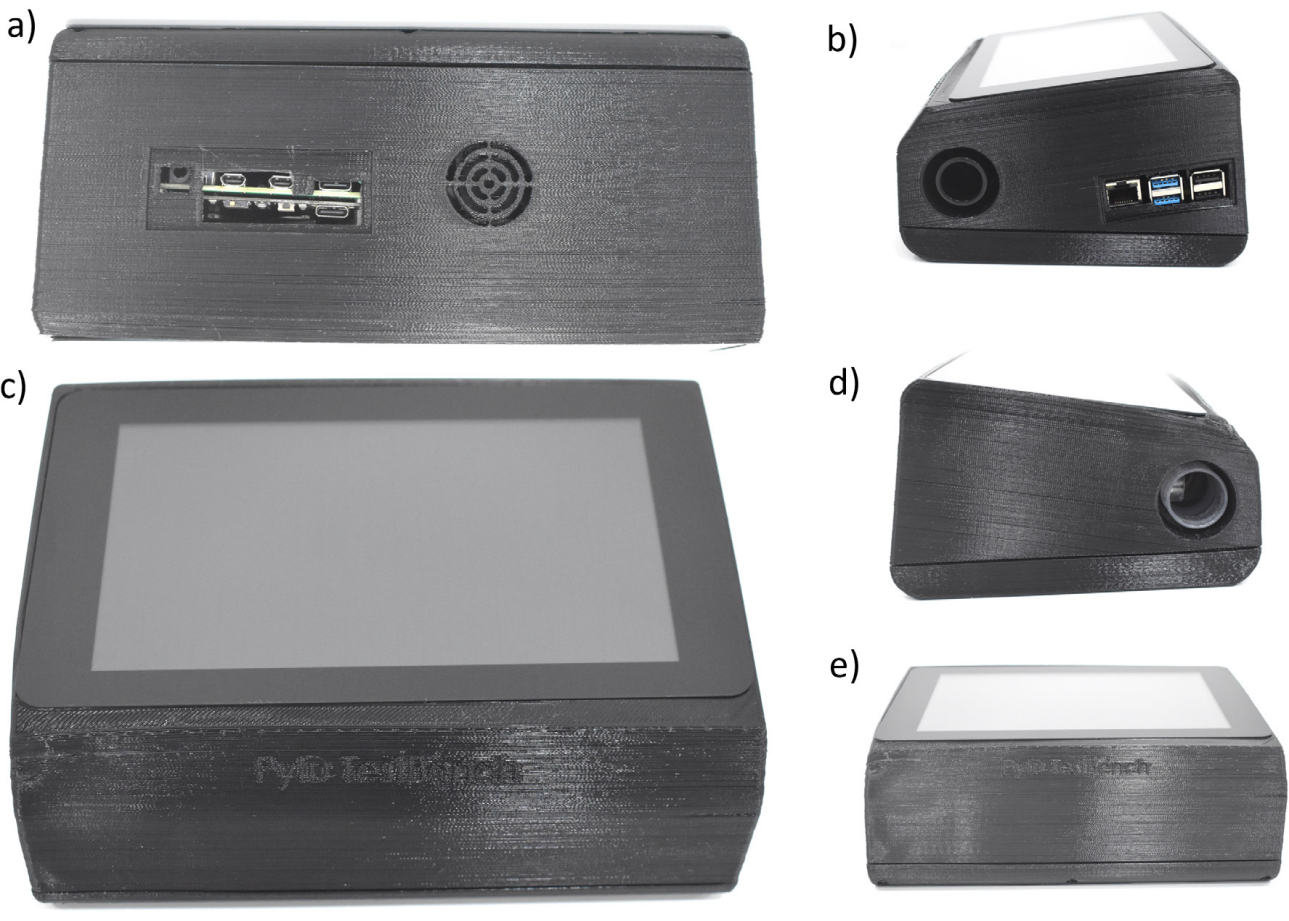
Assembly Part 3: (a) Back View, (b) Lateral view, (c) Top view, (d) Lateral view, (e) Frontal view.

### Software installation

PytuTester main program is written in python3 and requires a set of configurations and installation dependencies described below:

1. Initial setup to enable SPI, DSI output, and I2C interfaces.
2. Wirigpi: a PIN-based GPIO access library written in C for the BCM2835, BCM2836, and BCM2837 SoC devices used in all Raspberry Pi.
3. PypiADC: Python module for interfacing Texas Instruments SPI bus-based analog-to-digital converters with the Raspberry Pi.
4. Kivy: Open source Python library for rapid development of applications that make use of innovative user interfaces, such as multi-touch apps.
5. PytuTester App: Our program.

## Operation instructions

**Fig. 9:**
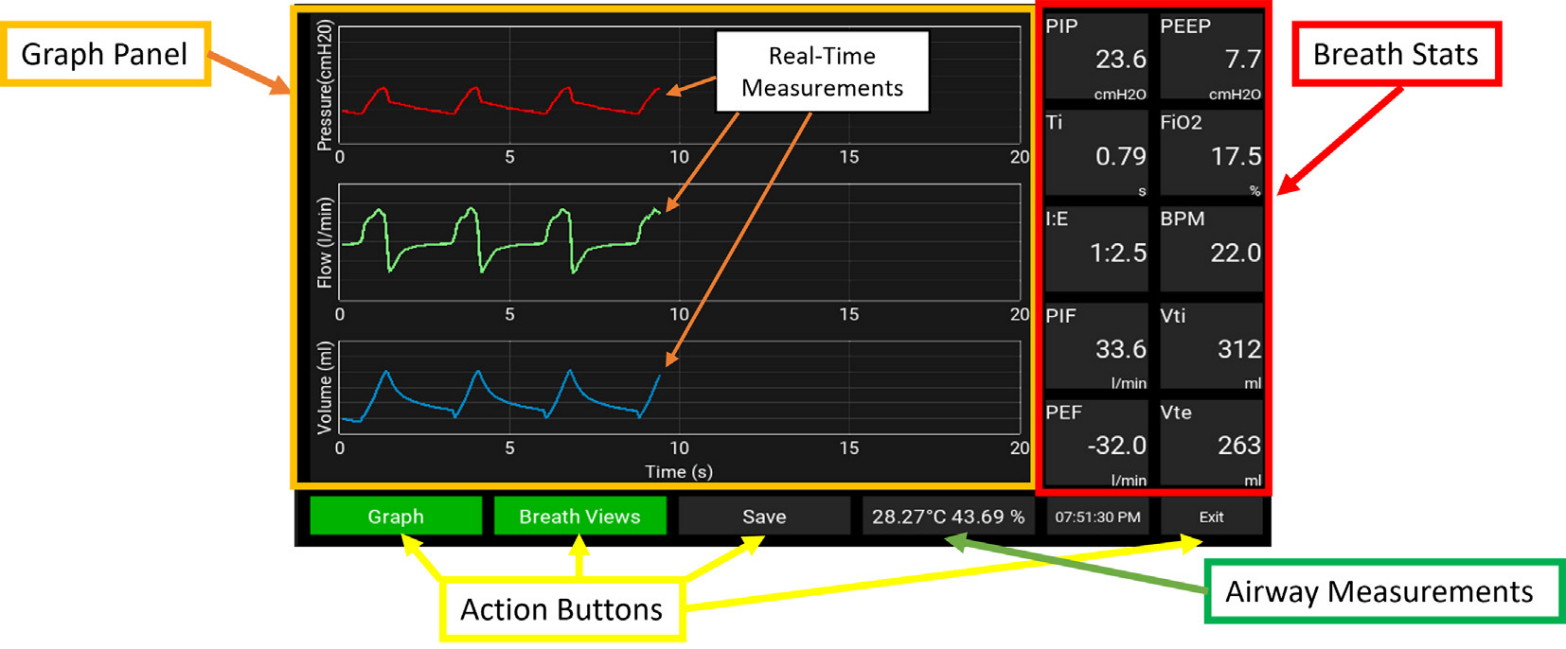
Graphical Interface.

The application developed that controls the DAQ runs on an operating system optimized for the Raspberry Pi hardware. The graphical interface GUI runs at SO startup. The GUI contains the graph panel, the breath views panel, and for action buttons assigned to start the real-time view (Graph), compute the stats (Breath View), the data-logger mode (Save), and exit. The GUI panels are divided into three main sections which are highlighted by different colors in Fig. 9.

**Fig. 10:**
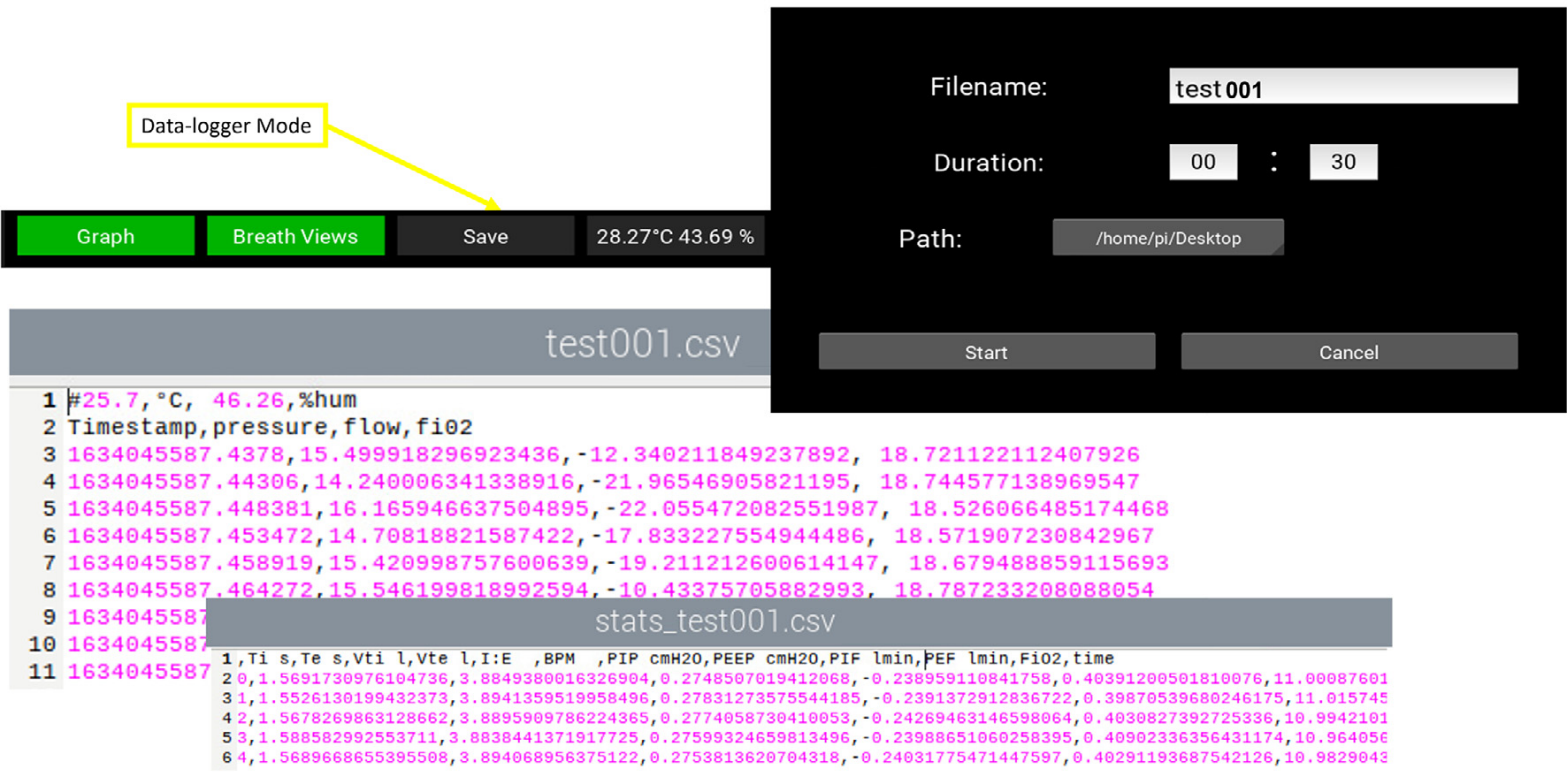
Data-logger Mode: Raw Data and Breath Stats Output.

The button Save opens a new window to set up the filename, path and the time save the raw measurements with a high sampling rate (> 200 sps). Fig. 10 shows the interface setup and the output files which are the raw data and the breath stats summary per each cycle. To avoid the exposition of air-borne viruses it is recommended to use a HEPA hydrophobic bacterial/viral filter.

## Validation and characterization

**Fig. 11:**
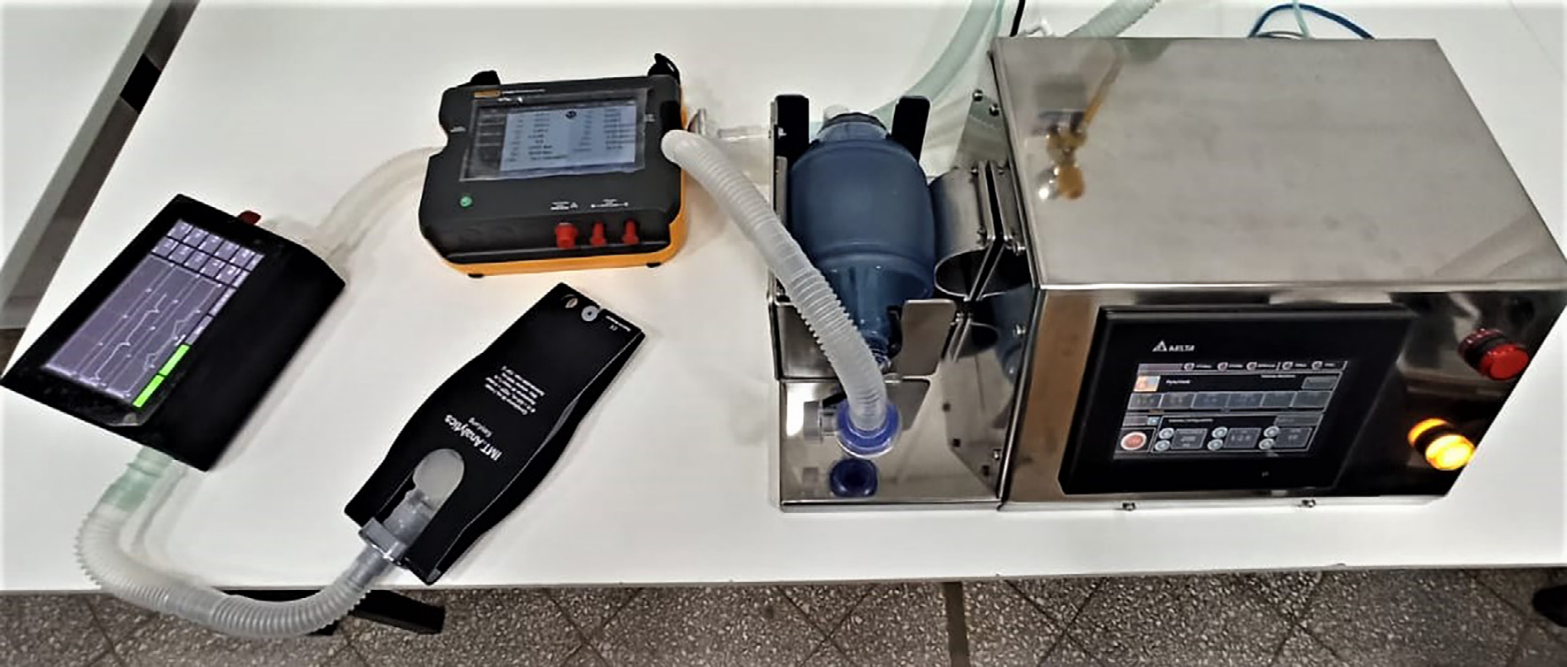
Validation Setup.

Our tester was designed to verify that ventilator prototypes meet the standard requirements; such as [4], [5]. For a Volume Control Inflation Type, the standards require the use of a calibrated flow and pressure sensor with a 10% to 90% rise time of no greater than 10 ms, temperature sensor, oxygen sensor with a data acquisition with a minimum sampling rate of 200 samples/s. This tester was used to measure variables of mechanical ventilator prototypes such as tidal volume, flow, pressure, and oxygen concentration [12]. The prototype output was connected to a reference ventilator tester, then our tester with a test lung for adults with 25 mL/mbar and 20 mbar/L/s values of compliance and resistance respectively^3^, as shown in Fig. 11. Our reference measurements were obtained with Fluke gas flow analyzer VT650. The validation test was performed for different ventilator setups varying the tidal volume ($V_{t}$), respiratory frequency (BPM), inspiration to expiration ratio (IE) to a set positive end-expiratory pressure (PEEP), and oxygen concentration FiO_2_, see Table 5. A power consumption test indicates that the current required by the system variate between 0.9 and 1 A, approximately 5 Watts. The lower values were obtained during the data logging mode, i.e. 0.9 A. The real-time view requires a little more, i.e. 1 A.

**Table 5:** Validation tests.

**Variable**	**Test**	**Setup**	**Fixed values**
$V_{t}$	1–4	200, 300, 400, 500 (mL)	IE = 0.5, BPM = 15 PEEP = 0,${\mathrm{FiO}}_{2}$=21%
PEEP	5–7	5, 10, 20 cm$H_{2}O$	$V_{t}$=300, IE = 0.5, BPM = 15,${\mathrm{FiO}}_{2}$=21%
(BPM,$V_{t}$)	8–9	(500, 20), (200, 10)	IE = 0.5, IE = 0.5, PEEP = 0, ${\mathrm{FIO}}_{2}$=21%
BPM	10–11	20, 12	$V_{t}$=300, IE = 0.5, PEEP = 0,${\mathrm{FiO}}_{2}$=21%
${\mathrm{FiO}}_{2}$	12–16	33%, 48%, 59%, 93%	- - -

**Fig. 12:**
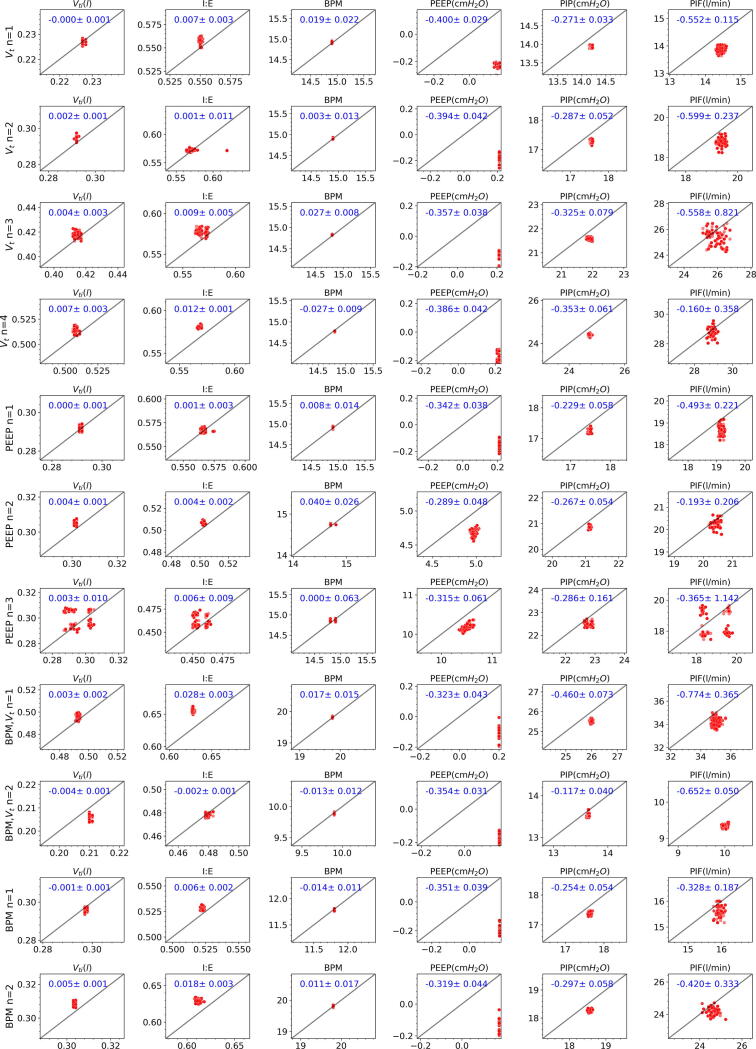
Scatter Plots.

Fig. 12 scatter plots panel presents PytuTester measurements (y-axis) as a function of the values measured (x-axis) by our calibrated gas flow analyzer. Each column presents the breath stats estimated values and a blue text indicates their corresponding means and standard deviation of the absolute errors during 3 min of measurements. Each row shows results for the combination of set values. The columns indicates that the mean largest errors are between (−4.0, 7.0) ml, (−0.0025, 0.028), (−0.027, 0.027), (−0.400, −0.289) cm$H_{2}O$, (−0.460, −0.117) cm$H_{2}O$ and (−0.774, −0.160) l/min for tidal volume, IE, BPM, PEEP, PIP, and PIF respectively. Rows 4 indicates that the PEEP set value can introduce a bias of 7 ml in tidal inspired volume. While rows 9 indicates that lower BPM can introduce a bias of −4 ml in tidal volume.

The tidal volume errors are reasonable considering that the VT650 has an error in the order of ±2.0% or 0.02 L, and we are only 6 mL from it in the worst case. During the real-time mode, the integration is performed by the forward Euler Method. While on the data-logger mode, this process is done during the postprocessing. The postprocessing applies several filters to reduce the noise. In this case, we use the composite Simpson’s rule to obtain more accurate results for the integration. All the scripts implemented are available on the software repository Metrics related to event reconstructions.

**Fig. 13:**
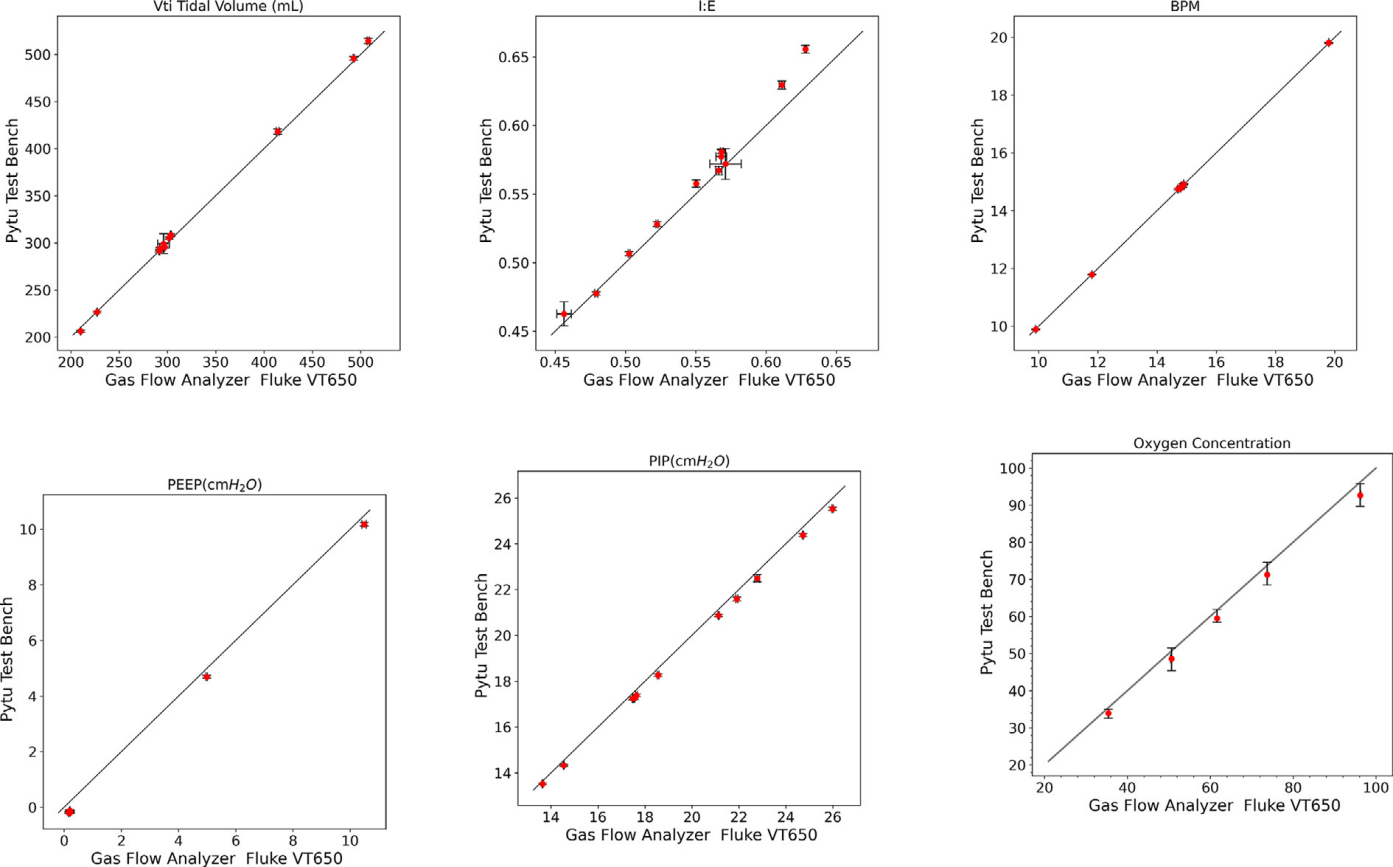
Validation Results.

Fig. 13 shows the combined tests scatter plots with an error bar for each test. Panel (a) indicates that when the tidal volume is estimated correctly. Panel (b) indicates that I:E can be slightly overestimated when the. Panels (c,d, and e) shows that the BPM, PEEP, and PIP values are estimated with high accuracy. Finally, Panel (f) indicates the FIO_2_ is slightly underestimated by our sensor with a mean absolute error between (−1.76, −3.52)%.

Table 6 summarizes absolute error during all tests performed. More tests can be done to implement bias correction functions using our validation test results. PytuTester errors are low enough to verify and evaluate new prototypes. Also, the pressure, flow, and volume curves obtained can provide information about the efficacy of the ventilator control systems.

**Table 6:** Estimated absolute errors.

**Variable**	**V**_**ti**_	**IE**	**BPM**	**PEEP**	**PIP**	**PIF**	**T**_**i**_	**T**_**e**_	**FiO**_**2**_
Absolute Errors	mL			cm$H_{2}O$	cm$H_{2}O$	l/min	s	s	%
Mean	2.970	0.009	0.016	0.348	0.286	0.463	0.012	0.012	2.528
Q_3_	3.856	0.011	0.023	0.372	0.311	0.578	0.017	0.017	2.62
Max	6.502	0.028	0.040	0.400	0.460	0.774	0.029	0.031	3.64

PytuTester software runs on a general purpose operating system, eg. Raspbian. This means that we do not have a real-time scheduler, and the sampled rate set on the ADC may run slower than expected. Additionally, the ads1256 driver adds some delays when reading and processing data in order to ensure stability and accuracy. We did an experiment to accurately evaluate the relation between the set values and the real sample rate. Table 7 shows that when we set a sample rate of 1000 sps (per channel), we can obtain 221 sps (when we measure three variables i.e., flow, pressure, and FiO2), which is required by the standard. Then during all validation tests, a set value of 1000 sps (per channel) was considered.

**Table 7:** Sampling Rate Verification.

**Set value: sps (p/ channel)**	500	1000	2000	3750
samples during 60s	7636	13285	20004	27092
sps (measured)	127.3	221.4	333.4	451.5

## Conclusion

The PytuTester is an open-source ventilator tester developed using readily available components. It was designed to measure the reliability, flow, pressure, volume, consistency of tidal volume, and Fi02 provided to the patient according to the ventilator standard, such as ISO80601-2-12. PyTu tester results were compared with measurements obtained using a calibrated Gas Flow Analyzer (VT650). The data acquisition system can read analog sensors with a sampling rate higher than 200 sps. The configurable operation modes help to evaluate the flow, pressure, and volume waveforms. The UPS system and its 3D structure guaranteed its portability. The software implements an extensible and easy-to-use graphical interface.

PytuTester hardware and software are public and can be improved to support several languages and more features. It will support the bioengineering capacity building especially in developing countries where there are not enough gas flow analyzers to verify and validate new ventilator prototypes. This new design must progress beyond some limitations; such as, the GUI which can be extended to support several languages, showing the battery level. The estimation algorithms should be adjusted according to standards definitions. The mechanical design can be improved to use less PLA or plastic injection molding. A low-cost, easily assembled ventilator testing platform could allow for the implementation of DIY patient care ventilators in low-resource settings.

## CRediT author statement

**Félix Morales**: Hardware and Software Design, Writing- Original draft **Luis Bernal**: Hardware Integration, Budget, and public repository. **Gustavo Pereira**: Validation. **D. H. Stalder**: Software testing, Improving GUI, Validation, Writing- Original draft preparation and supervision. **Sandra Perez and Michael Kammer**: Writing-Reviewing and Editing.

## Declaration of Competing Interest

The authors declare that they have no known competing financial interests or personal relationships that could have appeared to influence the work reported in this paper.

## Footnotes

1 http://kivy.org/.

2 http://github.com/ul-gh/PiPyADC.

3 IMT.analytics 1L Test Lung
